# Exploration of small molecules as inhibitors of potential BACE1 protein to treat amyloid cerebrovascular disease by employing molecular modeling and simulation approaches

**DOI:** 10.1371/journal.pone.0317716

**Published:** 2025-03-21

**Authors:** Zhizhong Wang, Zhiyong Li, Ailong Lin, Qing Zhang, Yingchun Chen, Bizhou Bie, Juanjuan Feng

**Affiliations:** 1 College of Chinese Medicine, Hubei University of Chinese Medicine, Wuhan, Hubei, China; 2 The Third People’s Hospital of Hubei Province, Affiliated Jianghan University, Wuhan, Hubei, China; Cukurova University: Cukurova Universitesi, TÜRKIYE

## Abstract

Amyloid cerebrovascular disease, primarily driven by the accumulation of amyloid-beta (Aβ) peptides, is intricately linked to neurodegenerative disorders like Alzheimer’s disease. BACE1 (beta-site amyloid precursor protein cleaving enzyme 1) plays a critical role in the production of Aβ, making it a key therapeutic target. In the current work, a CNS library of ChemDiv database containing 44085 compounds was screened against the BACE1 protein. Initially, a structure-based pharmacophore hypothesis was constructed, followed by virtual screening, with the screened hits docked to the BACE1 protein to determine the optimal binding modes. The docking results were examined using the glide gscore and chemical interactions of the docked molecules. The cutoff value of −5 kcal/mol was used to select hits with high binding affinities. A total of seven hits were chosen based on the glide g score. Furthermore, the possible binding mechanisms of the docked ligands were investigated, and it was discovered that all seven selected ligands occupied the same site in the predicted binding pocket of protein. The bioactivity scores of the compounds demonstrated that the chosen compounds possess the features of lead compounds. The toxicity risks and ADMET features of the selected hits were anticipated, and four compounds, **J032-0080**, **SC13-0774**, **V030-0915**, and **V006-5608** were chosen for stability analysis. The selected hits were extremely stable and strongly bound to the BACE1 pocket, and conformational changes caused by RMSD, RMSF, and protein-ligand interactions were assessed using MD modeling. Similarly, principal component analysis revealed a large static number of hydrogen bonds. The MM/GBSA binding free energies maps revealed a significant energy contribution in the binding of selected hits to BACE1. The binding free energy landscapes indicated that the hits were bound with a high binding affinity. Thus, the hits could serve as lead compounds in biophysical investigations to limit the biological activity of the BACE1 protein.

## 1. Introduction

Amyloid cerebrovascular disease refers to a collection of disorders characterized by the accumulation of amyloid-beta (Aβ) peptides in the walls of cerebral blood vessels, resulting in vascular impairment and heightened susceptibility to cognitive deterioration and hemorrhagic stroke [1]. Alzheimer’s disease (AD) is a progressive neurological ailment that causes cognitive decline and memory loss. Amyloid-beta (Aβ) peptides accumulate and form plaques in the brain, leading to neuronal dysfunction and cell death [2–4].

The enzyme β-site amyloid precursor protein cleaving enzyme 1 (BACE1) generates Aβ peptides by cleaving amyloid precursor protein (APP). BACE1 is a key therapeutic target for reducing Aβ generation and potentially slowing the progression of Alzheimer’s disease [5–8]. The Aβ1-40 peptide is notably associated with cerebral amyloid angiopathy (CAA), since it accumulates in the walls of cerebral blood vessels, impairing normal vascular function and integrity [9]. Amyloid deposits compromise artery wall integrity, increasing the risk of rupture and hemorrhage, perhaps leading to strokes or other serious neurological consequences [10].Targeting BACE1 to reduce Aβ levels is a viable technique to address the underlying mechanisms of Alzheimer’s disease and amyloid cerebrovascular diseases, potentially reducing the risk of related cognitive decline and cerebrovascular accidents [6,11,12].

In recent years, major efforts have been made to identify and produce small-molecule BACE1 inhibitors. These inhibitors try to reduce Aβ levels, slowing or stopping the course of AD. Computational techniques, such as molecular modeling and simulation, have been widely used to better understand the binding mechanisms of BACE1 inhibitors and to aid the development of more effective treatment drugs. For example, molecular dynamics (MD) simulations and binding free energy calculations have been used to examine the interactions between BACE1 and various inhibitors, giving insights into their binding affinities and stability [13–16].

Despite these advances, developing therapeutically effective BACE1 inhibitors has been difficult, with concerns such as blood-brain barrier permeability, off-target effects, and negative side effects. Notably, several BACE1 inhibitors in clinical trials were withdrawn due to safety concerns or a lack of efficacy. These losses highlight the need for alternative techniques and the discovery of new small compounds with better pharmacokinetic and pharmacodynamic properties.

The current study aims to solve these issues by using advanced molecular modeling and simulation tools to investigate and discover possible small-molecule BACE1 inhibitors. This study uses virtual screening, molecular docking, and MD simulations to unravel the binding processes and dynamic behaviors of new inhibitors, resulting in a thorough understanding of their therapeutic potential. This strategy not only improves drug discovery efficiency, but also allows for the identification of inhibitors with higher selectivity and fewer adverse effects.

## 2. Methodology

### 2.1. Structure refinement and validation

The crystal structure coordinates of the BACE1 (PDB ID: 2ZHV) were obtained from Protein Data Bank (https://www.rcsb.org/). There were some missing loop regions in the protein structure which were filled with the Modeller10.2 tool [17]. The modelled structure was then refined by the Galaxy web server (https://usegalaxy.org/) and then refined models were validated by the ERRAT, Verify 3D and PROCHECK tools of saves server (https://saves.mbi.ucla.edu/). PROCHECK examines the stereochemistry of protein structure and identifies the residues of the protein that require refinement by plotting Ramachandran plot. Ramachandran plot exhibited the residues reside in the allowed and disallowed regions.

### 2.2. Development of pharmacophore hypothesis

The refined structure of BACE1 protein was subjected to Maestro workspace. The structure was minimized and then the binding sites were selected by picking the co-crystal ligand. After binding sites selection, the receptor cavity based pharmacophore model was developed by Phase tool of Schrödinger 11.8:2018 [18]. The hypothesis was developed by choosing the receptor cavity binding pocket residues. Prior to developing the hypothesis, the receptor was prepared by adding the hydrogen atoms, and by fixing the side chain residues. The minimization of the receptor was done and then it was subjected to the structure-based hypothesis development.

### 2.3. Compound library preparation and virtual screening

The CNS library of ChemDiv database containing 44085 compounds was retrieved and processed for database preparation through Phase [18]. During preparation, the chemical space search was increased by generating twenty conformers of each compound at pH 7.0 by Epik [19]. Additionally, the high energy tautomer was removed. After preparing the database, the developed pharmacophore hypothesis was employed for virtual screening. The output of the screening was analyzed by phase screen score. The phase screen score is a combination of RMSD site matching, volume score, and vector alignments. The hits that met with the selection criteria of 1.4 phase score were selected for the molecular docking studies.

### 2.4. Molecular docking studies

The refined structure of BACE1 protein was processed for the preparation for molecular docking preparation wizard [20]. The preparation involved three steps: preprocessing, optimization, and minimization. In the preprocessing, the hydrogen atoms were added, extra chains were removed, the charges were added, and the side chain atoms of residues were fixed. Further, the tautomeric states were generated by PROPKA [21]. The hydrogen atoms were optimized at pH 7.0. and then the energy of structure was minimized by using OPLS2005 forcefield [22]. The processed structure was subjected for grid generation by selecting the co-crystal ligand. The X, Y, and Z coordinates of the generated grid were 75.17, 36.03, and 20.06, respectively. Further, the screened hits were prepared by LigPrep tool [23] and then by using the standard precision mode of Glide tool, docked to the prepared structure. The binding affinities of the docked compounds were calculated in terms of glide scores and the compounds with best glide scores were selected for further study.

### 2.5. Prediction of bioactivity scores

The biological activity of the selected compounds was predicted by using the Molinspiration tool (https://www.molinspiration.com/). The bioactivity of a drug can be impacted by the chemical structure of compound, its potency and lastly the selection of target. A potent drug candidate is expected to bind with the target enzyme or receptor with stability and it will deliver the therapeutic benefits after binding with the receptors and produce fewer side effects as compared to the compounds with lower potency [24]. The bioactivity scores of the compounds selected at the docking steps were predicted against the GPCR, the kinase proteins, several ion channels, proteases, and the inhibitors of enzymes.

### 2.6. ADMET analysis

The higher rate of drug erosion is often attributed to issues related to toxicity and poor pharmacokinetics [25]. To address these challenges, ADMET properties are predicted to assess the pharmacokinetic properties and toxicity risks associated with potential drug candidates [26]. This predictive approach also helps in evaluating the likelihood of lead compounds becoming viable oral drugs. In this study, we used OSIRIS Property Explorer tool [27] and SwissADME to predict the ADMET characteristics of the most promising compounds. Numerous pharmacokinetic characteristics were examined, such as molecular weight, Hydrogen bond acceptors, Hydrogen bond donors, Blood Brain Barrier permeability, CNS permeability, drug-likeness, hydrophilicity (log P), solubility (log S), and topological polar surface area (TPSA). Additionally, we scrutinized the compounds for potential toxicity risks, encompassing tumorigenic, mutagenic, irritating, and reproductive concerns.

### 2.7. MD simulation

To analyze the stability of complexes, an MD simulation for 100 ns was executed. The solvation of the complex was done in a periodic box with a 10 Å size containing the TIP3P water molecules [28]. Counter ions of Na + and Cl− were introduced into the system to neutralize it. The minimization of the system was performed using the steepest decent method of 5000 steps following neutralization to remove steric conflicts. After minimization, the systems were prepared for the production run by equilibrating for 50,000 and 100,000 steps, respectively, at 310 K temperature at the NVT and NPT ensembles [29]. The simulation was Conducted with the Berendsen thermostat and Parrinello-Rahman algorithms to maintain a constant temperature (310 K) and pressure (1 atm). By adjusting the time at τ P = 2.0 ps and τ T = 0.1 ps, the system was relaxed, and by applying the LINCS algorithm, the hydrogen atoms’ bond lengths were kept at their ideal lengths [30], whereas Verlet computed the non-bonded interactions [31]. To compute the electrostatic interactions beyond the short-range limit, the particle mesh Ewald approach was used [32]. In x, y, and z dimensions, the Periodic boundary conditions were imposed, and a production run was conducted on the system. Every 10 ps, the production run’s trajectory was saved and examined using the R BIO3D package and gromacs commands [33]. CHARMM36 forcefield and the Gromacs simulation program were used to execute the simulation [34].

### 2.8. Binding free energy calculations

The binding free energy estimations obtained by the MM/GBSA approach are more reliable than those obtained by numerous molecular docking scoring systems [35,36]. Similarly, several studies have demonstrated that the MM/GBSA approach is sufficiently precise and stable to predict the binding free energies between protein targets and small drug-like compounds [37,38]. Using the MM/GBSA approach and the equation below, the binding free energies of the complexes were determined while keeping these findings in mind.


$$\Delta G_{\text{bind}}=\Delta G_{comp}-\Delta G_{pro}-\Delta G_{lig}$$

## 3. Results

### 3.1. Structure evaluation

**Fig 1:**
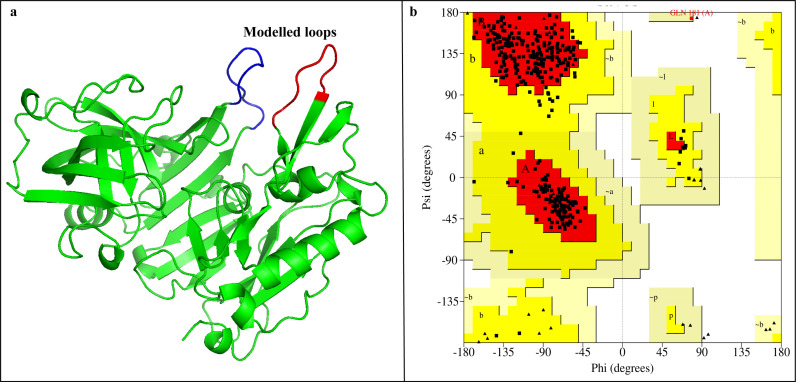
(a) The modelled structure of BACE1 protein. The green region shows the original structure while the blue and red regions depict the modelled residues. (b) The Ramachandran plot indicates the presence of most of the residues in the favored regions (red).

The crystal structure of the BACE1 protein was retrieved from Protein Data Bank. The structure was analyzed and there were missing residues in the loops. The missing loops were modelled by the Modeller tool. Fig 1a shows the modelled part of the loops. Green cartoon structure indicates the original crystal structure while blue and red loops show the modelled structure. After the modelling, the structure was refined by the Galaxy refined tool. It generated five models, and the qualities of the refined models were evaluated by the ERRAT, Verify 3D, MolProbity, and PROCHECK tools. The parameters are shown in Table 1. Among the refined models, model 2 showed better quality than the others, so it was selected for further study. The Ramachandran plot of model 2 is shown in Fig 1b. It shows the percentage of amino acid residues reside in the allowed regions. The Ramachandran plot of model 2 showed that there were 338 residues (98%) in the favored region (red), 11 residues in the additional allowed region (yellow), while no residue in the disallowed region (white).

**Table 1:** The models generated by galaxy refine tool along with the evaluation parameters.

Model	GDT-HA	RMSD	MolProbity	Clash score	Poor rotamers	Rama favored	ERRAT	Verify 3D
1	0.977	0.33	1.78	15.5	0.6	97.6	88.54	Pass
2	0.976	0.33	1.83	16.5	1.4	98.0	93.63	Pass
3	0.973	0.34	1.66	14.5	0.9	98.3	89.26	Pass
4	0.976	0.33	1.68	13.5	1.1	98.0	90.12	Pass
5	0.976	0.34	1.80	16.2	0.9	97.6	90.60	Pass

### 3.2. Pharmacophore hypothesis development

**Fig 2:**
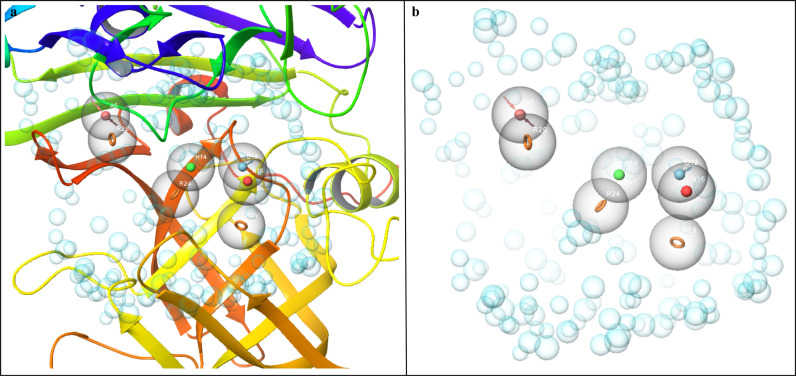
The developed pharmacophore models. (a) The pharmacophoric features shown in the protein pocket. (b) The receptor cavity along with the pharmacophoric features.

The refined structure of BACE1 was subjected to the workspace of Maestro and optimized for pharmacophore model development. The binding pocket residues were selected to generate the pharmacophore hypothesis containing a total of seven features. The pharmacophore hypothesis contained seven features, D (Hydrogen Bond Donor), H (Hydrophobic group), R (Aromatic Ring), N (Negative Ionizable group), A (Hydrogen Bond Acceptor), R (Aromatic Ring), and R (Aromatic Ring) (Table 2) shown in Fig 2a–b. As the binding site residues, i.e., Leu48, Asp50, Gly52, Ser53, Tyr89, Lys125, Phe126, Trp133, Ile136, Ile144, Arg146, Tyr216, Ile244, Asp246, Gly248, Thr250, and Arg253 were selected during the hypothesis generation, so the pharmacophoric features were generated based on these residues. These seven features were generated due to the high phase scores, so these were used in the virtual screening. The chemical compounds from the database matching at least 5 of the 7 features were selected.

**Table 2:** The pharmacophore hypothesis features along with scores and coordinates in the receptor cavity.

Rank	Features	Scores	X	Y	Z
1	D (Donor)	−2.2	64.87	49.52	10.36
2	H (Hydrophobic)	−1.89	68.09	48.12	7.64
3	R (Aromatic)	−1.61	70.58	48.80	7.97
4	N (Negative group)	−1.61	65.23	48.93	12.65
5	A (Acceptor)	−1.41	70.13	41.13	3.66
6	R (Aromatic)	−1.35	68.37	52.88	11.86
7	R (Aromatic)	−1.3	70.98	41.91	5.45

### 3.3. Virtual screening of CNS library

The developed pharmacophore model was employed for the virtual screening of CNS library of the ChemDiv database. During screening, a compound that matched at least four features among the seven pharmacophoric features, was selected as a hit. The screened hits were ranked by the phase screen score which is a combination of Volume score, RMSD site matching, and vector alignments. For better alignment, the vector score must be high in the range of −1.0 to 1.0. Similarly, the reference range for volume is 0.0 to 1.0 where high score shows the greater overlaps among the volume of reference ligand and aligned ligands. It is calculated by dividing the volume of aligned ligands by the volume of the total volume of two ligands, a zero score shows no reference ligand. The potential hits were identified by setting a cutoff phase screen score of 1.4. The compounds with 1.4 phases scores are given in Table 3.

**Table 3:** The hits generated during virtual screening, selected based on the phase screen scores.

No.	ChemDiv IDs	Phase scores	No.	ChemDiv IDs	Phase scores
1	V029-4368	1.58	46	SA67-1047	1.468
2	V027-7193	1.57	47	SC13-0774	1.467
3	V014-2412	1.547	48	T161-0081	1.467
4	V031-1735	1.533	49	Y044-5074	1.466
5	V014-2412	1.521	50	V013-5803	1.466
6	P636-0553	1.519	51	P891-1703	1.466
7	E957-0272	1.517	52	S751-0051	1.465
8	G620-0076	1.512	53	F426-0184	1.465
9	G620-0474	1.511	54	S779-0696	1.465
10	ZC43-0231	1.506	55	SC06-0583	1.464
11	V030-0915	1.502	56	K940-1353	1.463
12	8008-8428	1.501	57	CM4495-5106	1.463
13	SB52-0600	1.498	58	SB52-0124	1.461
14	S637-0006	1.493	59	C174-0271	1.461
15	S654-0054	1.493	60	L741-3275	1.461
16	7472-0036	1.489	61	D640-0002	1.46
17	K780-0305	1.489	62	D640-0002	1.46
18	V012-1864	1.487	63	G547-0107	1.46
19	8019-4421	1.487	64	P937-3939	1.459
20	L928-0416	1.486	65	C618-0390	1.458
21	L827-1858	1.486	66	L741-0071	1.457
22	SB24-0560	1.484	67	SA21-0515	1.457
23	D430-2300	1.484	68	P349-3424	1.456
24	SB32-0111	1.483	69	SA66-0867	1.456
25	G620-0296	1.483	70	V012-1886	1.456
26	SD02-0254	1.483	71	D672-0204	1.456
27	S248-3535	1.482	72	T655-0597	1.456
28	J032-0080	1.481	73	L741-3174	1.455
29	V013-5814	1.481	74	E958-0426	1.455
30	SC13-0120	1.479	75	SB52-1213	1.454
31	P937-3939	1.479	76	Y501-4997	1.453
32	T501-1335	1.478	77	P937-3939	1.453
33	Y041-8702	1.477	78	V006-5608	1.452
34	SC13-0120	1.477	79	Y206-6120	1.452
35	Y042-6425	1.474	80	SB52-1161	1.452
36	V014-2333	1.473	81	3381-0875	1.452
37	G547-0689	1.472	82	L741-0259	1.452
38	8013-2958	1.471	83	P891-1703	1.451
39	G620-0076	1.47	84	S564-0769	1.451
40	F255-0678	1.47	85	V017-8952	1.45
41	T408-2164	1.469	86	P349-3440	1.45
42	F255-0678	1.469	87	Y020-2597	1.45
43	S926-0952	1.469	88	S779-0988	1.45
44	T161-1027	1.468	89	P349-2041	1.449
45	V029-4368	1.58	90	F082-0164	1.449

### 3.4. Molecular docking

**Fig 3:**
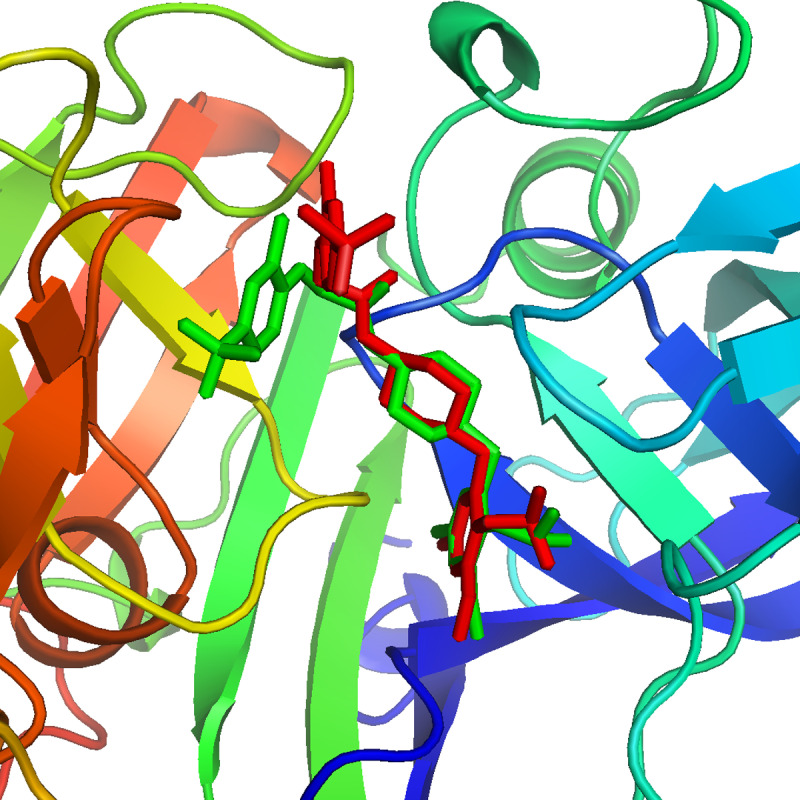
The native (green) and redocked pose (red) of co-crystal ligand in the binding pocket of BACE1 protein.

**Fig 4:**
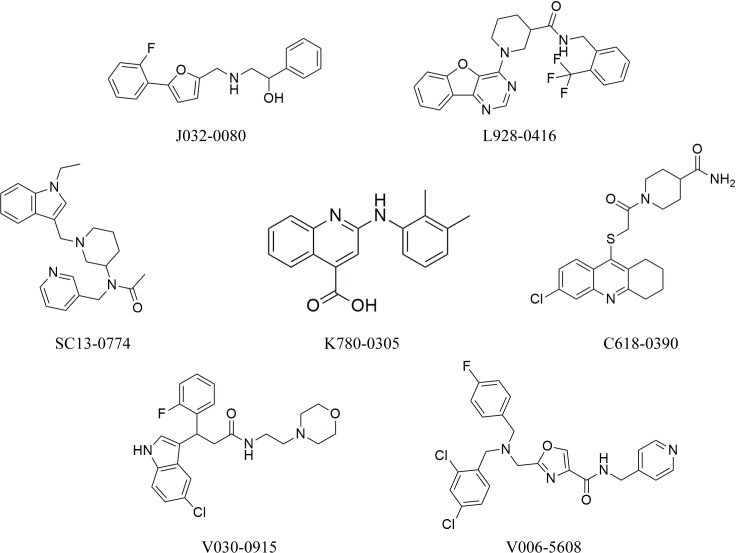
The molecular structures of the compounds selected based on the binding affinities.

**Fig 5:**
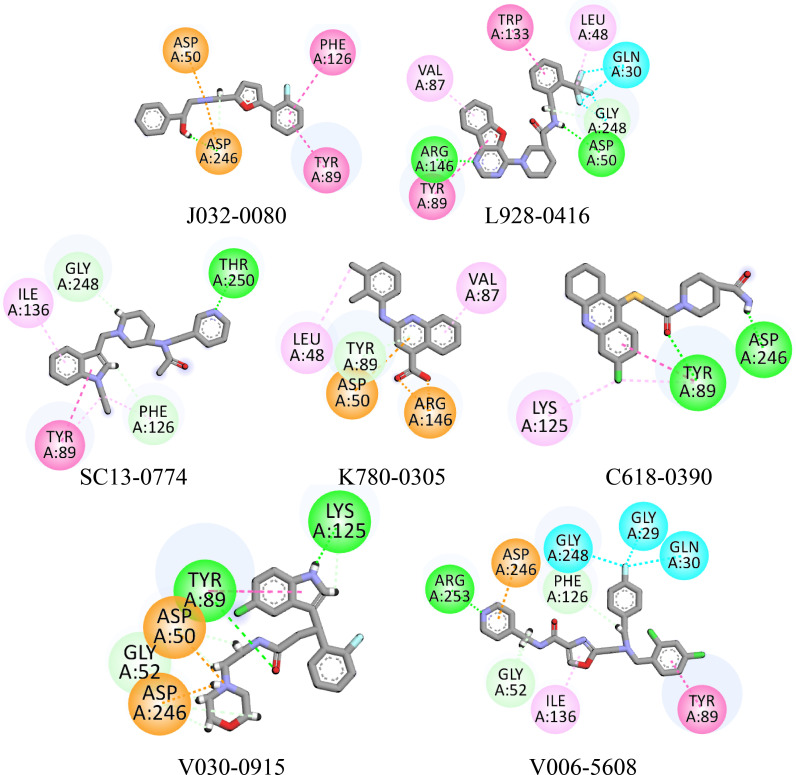
The binding interactions of hits with protein. The hydrogen bonds are shown by green spheres, hydrophobic interactions are shown by magenta color, the purple lines show pi-sigma, while orange lines show the pi-sulfur interactions.

**Fig 6:**
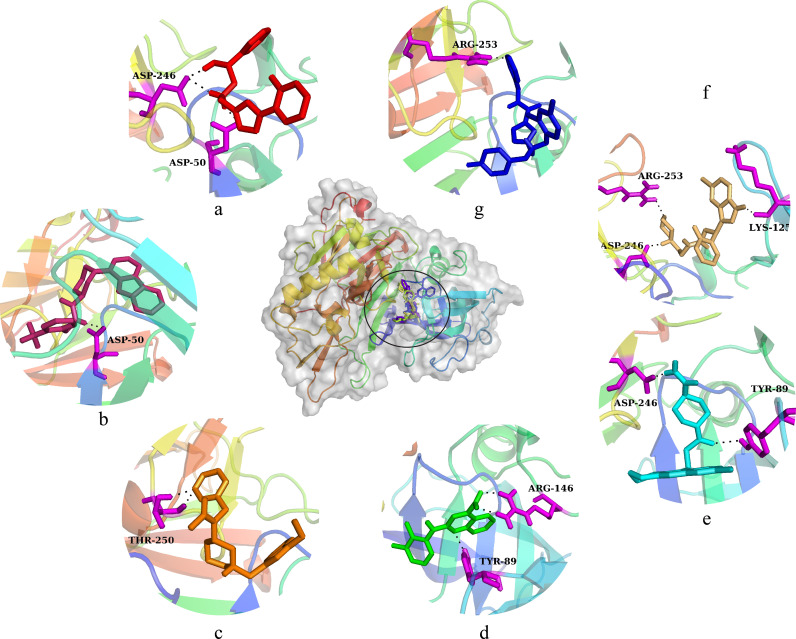
The plausible binding modes of the selected compounds are represented with the sticks in the binding pocket of protein. The orientation of the hits is also shown in the pocket separately. (a) J032-0080, (b) L928-0416, (c) SC13-0774, (d) K780-0305, (e) C618-0390, (f) V030-0915, (g) V006-5608.

The hits generated during the virtual screening were prepared and docked to the BACE1 receptor by SP docking module of glide. Prior to the docking of screened hits, the docking efficiency of the tool was evaluated by redocking of co-crystal ligand of BACE1 (PDB ID:2ZJN). The crystal ligand was redocked to the protein and the docked pose was aligned on the native pose. On alignment, it was observed that the redocked pose had a deviation of 0.5 with native pose (Fig 3). Hence, the docking of screened hits was performed, and the docking poses were analyzed based on the glide gscore and molecular interactions of the docked compounds. The compounds with binding affinities greater than −5 kcal/mol were selected for further analysis. The two-dimensional structures of the selected hits are shown in Fig 4. The molecular interactions of the selected docked hits were analyzed, and it was observed that **J032-0080** made two hydrogen bonds with Asp246 with 1.62 Å distance. **L928-0416** made three hydrogen bonds with Arg146, Asp50, and Gly248. **SC13-0774** and **K780-0305** engaged in hydrogen bonding with Gly248, Phe126, Thr250, and Tyr89. Similarly, **C618-0390** made two hydrogen bonds with Tyr89 and Asp246. **V030-0915** made four hydrogen bonds with Tyr89, Gly52, Asp246, and Lys125 while **V006-5608** engaged in hydrogen bonding with the residues Arg253, Gly52, and Phe126 (Fig 5). The docking scores, interacting residues, interaction types, and the distance between ligand and hydrogen bond forming residues are given in Table 4. Further, the plausible binding modes of the docked ligands were analyzed, and it was observed that all seven selected ligands occupied the same space in the predicted binding pocket of protein (Fig 6).

**Table 4:** The docking scores, interacting residues and the hydrogen bond distances of the selected compounds with protein.

ChemDiv IDs	Glide scores	Interacting residues	Types of interactions	Distance (Å) H-bonds
J032-0080	−6.345	Asp50, Asp246, Phe126, Tyr89	Hydrogen Bond, Pi-Sulfur, Alkyl, Pi-Alkyl	1.62, 3.0
L928-0416	−6.225	Val87, Tyr89, Arg146, Asp50, Gly248, Gln30, Leu48, Trp133	Hydrogen Bond, Pi-Cation, Alkyl, Pi-Alkyl, Halogen	2.25, 2.75, 2.83
SC13-0774	−6.108	Ile136, Tyr89, Gly248, Phe126, Thr250	Hydrogen Bond, Alkyl, Pi-Alkyl	3.61, 2.49, 3.04
K780-0305	−5.991	Leu48, Tyr89, Asp50, Arg146, Val87	Hydrogen Bond, Pi-Cation, Alkyl, Pi-Alkyl	2.78
C618-0390	−5.899	Tyr89, Lys125, Asp246	Hydrogen Bond, Alkyl, Pi-Alkyl	1.56, 2.55
V030-0915	−5.721	Gly52, Asp50, Tyr89, Lys125, Asp246	Hydrogen Bond, Salt Bridge, Alkyl, Pi-Alkyl	3.00, 1.88, 2.37
V006-5608	−5.675	Gly52, Ile136, Tyr89, Gln30, Gly29, Gly248, Phe126, Asp246, Arg253	Hydrogen Bond, Alkyl, Pi-Alkyl, Halogen	2.00, 2.58, 2.51

### 3.5. Bioactivity scores

The bioactivity scores of the compounds help to analyze the drug binding ability to various human receptors. The bioactivity scores of the selected seven compounds were predicted by Molinspiration tool. The predicted scores of the compounds against several receptors are shown in Table 5, indicating that the scores were in the range of −5 to 0.0. showing the moderate activity of these compounds against the receptors. Some compounds showed scores close to 0, indicating good activity against the receptors. The analysis revealed that the selected compounds have the properties of lead compounds.

**Table 5:** The predicted bioactivity scores of the selected compounds against different human receptors.

ChemDiv IDs	GPCR ligand	Ion channel modulator	Kinase inhibitor	Nuclear receptor ligand	Protease inhibitor	Enzyme inhibitor
J032-0080	−0.06	−0.33	−0.04	−0.19	−0.22	−0.01
L928-0416	−0.15	−0.19	−0.02	−0.42	−0.20	−0.02
SC13-0774	−0.34	−0.05	−0.17	−0.26	−0.13	−0.14
K780-0305	−0.01	−0.02	−0.27	−0.01	−0.26	−0.07
C618-0390	−0.08	−0.35	−0.40	−0.59	−0.17	−0.01
V030-0915	−0.14	−0.19	−0.13	−0.35	−0.07	−0.17
V006-5608	−0.08	−0.17	−0.10	−0.38	−0.05	−0.02

### 3.6. ADMET analysis

The selected toxicity risks and ADMET properties of the selected hits were predicted using OSIRIS Property Explorer and SwissADME. The molecular weight of a compound indicates its easy distribution in the cells so the compounds with less weight can easily distribute in the body as compared to the compounds with higher weight. In this regard, a criterion of 500 g/mol was set, and all the molecular weights of all selected compounds fall within this range. cLogP determines the hydrophilicity of compound, a value of cLogP > 5 indicates the low hydrophilicity or poor absorption. The selected hits had cLogP values less than 5, indicating good absorption of compounds. The TPSA (Topological Polar Surface Area) relates with the hydrogen bonding of a compound and is a good predictor of bioavailability [39]. TPSA < 160 Å^2^ shows that the compound will have good oral bioavailability [40]. The hits had TPSA values in the range of 39.5 to 128.1 Å^2^. Solubility is also a crucial factor in pharmacokinetics, influencing both the absorption and distribution of a compound. It is typically quantified as the logarithm of the solubility, expressed in mol/dm^3^. This measurement helps to assess how easily a compound dissolves in a solvent, which is vital for its effective utilization in the body and its overall pharmacokinetic profile. Similarly, a positive score for drug-likeness suggests that the molecule shares common structural features with existing drugs, making it a more promising candidate for drug development. All hits had positive drug-likeness. The drug score is a thorough evaluation that combines several properties, such as drug-likeness, cLogP, logS, molecular weight, and toxicity risk, into a single, understandable value. The overall potential of a molecule to be developed into a medication is assessed using this score. A chemical is more likely to be a good option for therapy if it has a higher drug score. In other words, a molecule with a higher drug score value is more likely to be selected for additional therapeutic research [41]. The hydrogen bond donors and acceptors were also in the acceptable range, i.e., HBA < 10 and HBD < 5. Further, the permeability of Blood Brain Barrier and CNS was estimated, and it was observed that two compounds **L928-0416** and **C618-0390** did not have the BBB permeability while other compounds can pass it. In the case of CNS permeability, all compounds have this ability (Table 6). Furthermore, the toxicity profile of compounds was evaluated, and it was observed that the compounds did not show toxicity tendencies except for **K780-0305** and **C618-0390** which have high risk of irritant and mutagenicity respectively (Table 7). Based on the analysis, four compounds **J032-0080**, **SC13-0774**, **V030-0915**, and **V006-5608** were selected for the stability analysis.

**Table 6:** The pharmacokinetic property and druglikeness analysis of the selected hits.

IDs	MW	HBA	HBD	cLogP	TPSA	LogS	BBB	CNS	Druglikeness	Drugscore
J032-0080	311	4	2	2.83	45.4	−4.15	Yes	Yes	2.95	0.75
L928-0416	454	7	1	4.38	71.26	−6.41	No	Yes	−6.29	0.2
SC13-0774	390	3	0	2.69	41.37	−2.32	Yes	Yes	5.45	0.83
K780-0305	292	3	2	3.86	62.22	−5.13	Yes	Yes	−0.46	0.26
C618-0390	417	3	1	3.44	101.5	−5.03	No	Yes	1.09	0.31
V030-0915	429	4	2	3.49	57.36	−4.07	Yes	Yes	1.53	0.61
V006-5608	498	6	1	3.8	71.26	−4.85	Yes	Yes	4.92	0.71

**Table 7:** The Toxicity risks analysis of the selected hits.

ChemDiv IDs	Mutagenic	Tumorigenic	Irritant	Reproductive effect
J032-0080	Passed	Passed	Passed	Passed
L928-0416	Passed	Passed	Passed	Passed
SC13-0774	Passed	Passed	Passed	Passed
K780-0305	Passed	Passed	High risk	Passed
C618-0390	High risk	Passed	Passed	Passed
V030-0915	Passed	Passed	Passed	Passed
V006-5608	Passed	Passed	Passed	Passed

### 3.7. MD simulation

After the ADMET analysis, four compounds with highest drug-likeness and not toxicity risks were selected for the stability analysis by conducting 100 ns simulation. The MD trajectories were analyzed by measuring the RMSD, RMSF, Radius of gyration, Hydrogen bonding, PCA, and binding free energy calculations.

**Fig 7:**
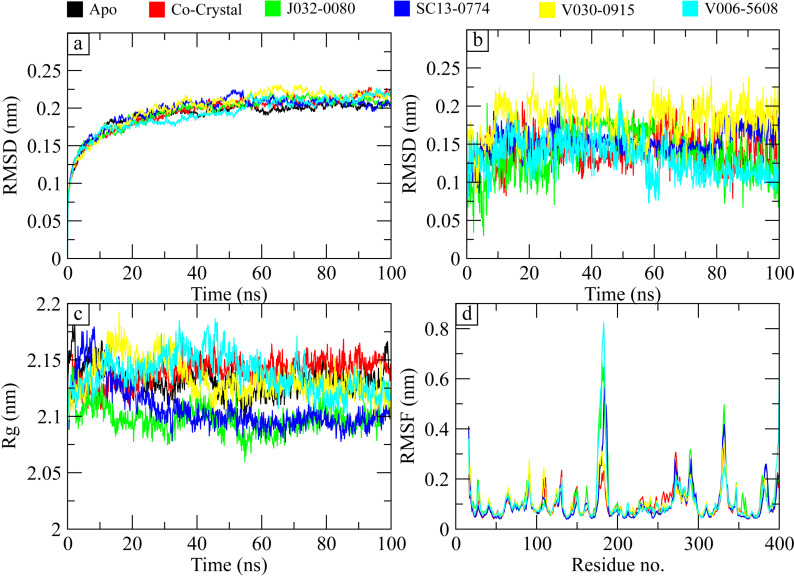
The protein-ligand complex stability analysis during simulation. (a) The RMSD comparison of apo protein with protein complexes. (b) The RMSDs of the ligands. (c) The Rg analysis. (d) The residual flexibility of the apo protein and complexes.

The RMSD of carbon alpha atoms of protein in apo form was calculated compared with the protein complexes to investigate the stability of protein ligand complex [42]. It can be observed that the RMSD of apo protein gradually increased to 0.2 nm at 20 ns and showed deviations in the range of 0.2–0.25 nm till 100 ns. The RMSD of co-crystal ligand also showed the same trend in the RMSD values as showed by the apo protein. Similarly, the RMSD values of the hit complexes showed the same trend except for some minor deviations in the **SC13-0774** and **V030-0915** complexes where RMSD values increased to 0.25 nm for some time but again attained the stable values shown by the apo protein. The RMSD analysis showed that the binding of ligands did not exert conformational changes to the protein structure, and they remained stably bound to the protein (Fig 7a). Further, the RMSDs of the ligands were calculated and it was observed that the RMSD of all ligands remained less than 0.2 nm throughout the simulation (Fig 7b). Similarly, the radius of gyration was calculated to find the compactness of protein structure upon binding of the ligands. The Rg values show the compactness of structure, higher the value, lower the compactness. From the Rg graph, it can be observed that the initial Rg value of apo protein was around 2.15 nm which reduced to 2.12 nm at 50 ns and then remained in this range till the end of simulation. The Rg values of the complexes was compared to the apo protein, and it was observed that the Rg values of **J032-0080** and **SC13-0774** were lower than the apo protein, the Rg values of **V006-5608** were slightly higher than apo protein while the Rg values of **V030-0915** were like apo protein in second half of simulation. The small deviations in the Rg values indicated the compactness of protein structure upon binding of the hits (Fig 7c). Root mean square fluctuations (RMSF) values have been calculated in order to examine the fluctuating behavior of the proteins while they are bound to the ligands [43]. For each protein residue over the simulation period, RMSF values give detailed information on the residue’s flexibility and mobility. Based on the expected RMSF values, most protein residues changed very slightly during the simulation, measuring less than 0.2 nm except for the large loop region ranging from 170 to 190 residues. The loop region also showed higher RMSF values in apo protein, so it suggests that the residues that form the contacts with the ligand did not undergo any confirmational changes and remained stably bound with the ligands (Fig 7d). Overall, RMSF values are compatible with the idea of a stable protein and ligand.

**Fig 8:**
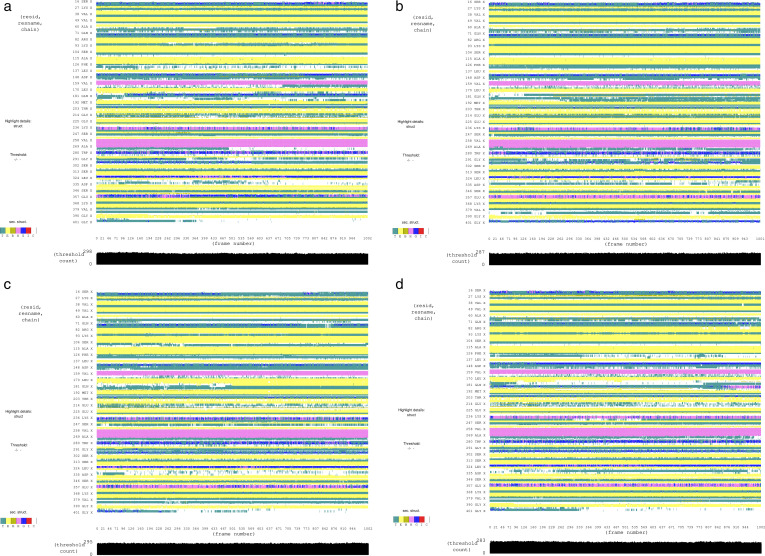
The secondary structure analysis of the complexes during simulation. a = J032-0080, b = SC13-0774, c = V030-0915, d = V006-5608.

**Fig 9:**
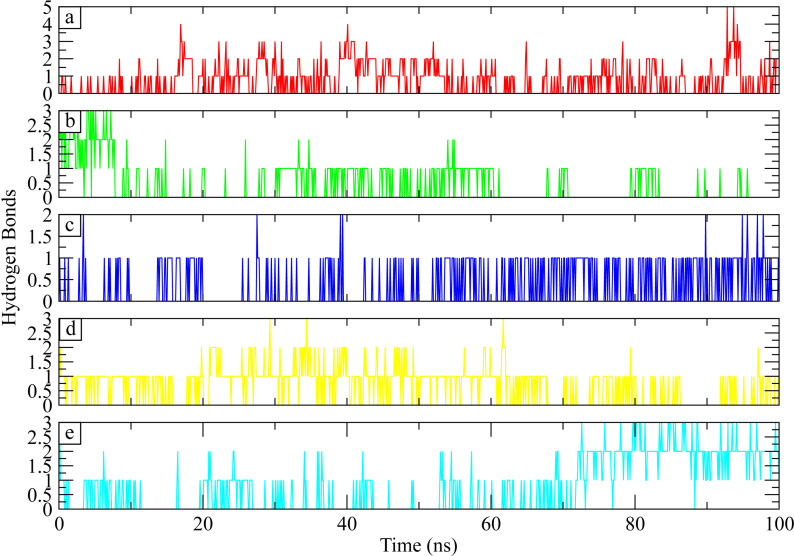
The number of hydrogen bonds formed by the hits with protein during simulation. a = Co-crystal ligand, b = J032-0080, c = SC13-0774, d = V030-0915, e = V006-5608.

To analyze the difference between the complexes of the BACE1 protein, secondary structural changes were observed throughout the simulation. The basic secondary structures were denoted by different letters, i.e., T (Turn), E (Beta Sheet), B (Beta Bridge), H (Helix), G (3-10 Helix), I (Pi Helix), and C (Loop). From the graphs, it can be observed that the helices and beta sheets were similar in all four complexes during the simulation. However, the number of turns were different especially in case of **SC13-0774** complex where the turns were more than other complexes (Fig 8). Similarly, Hydrogen bond analysis was conducted which is essential for the protein-ligand stability. Consequently, the number of hydrogen bonds of ligands with the active site residues were calculated. The hydrogen bonding plots indicate that **Co-crystal ligand**, **V030-0915** and **V006-5608** made at least 2 hydrogen bonds with the protein while the remaining two complexes showed one hydrogen bond at most of the frames (Fig 9).

**Fig 10:**
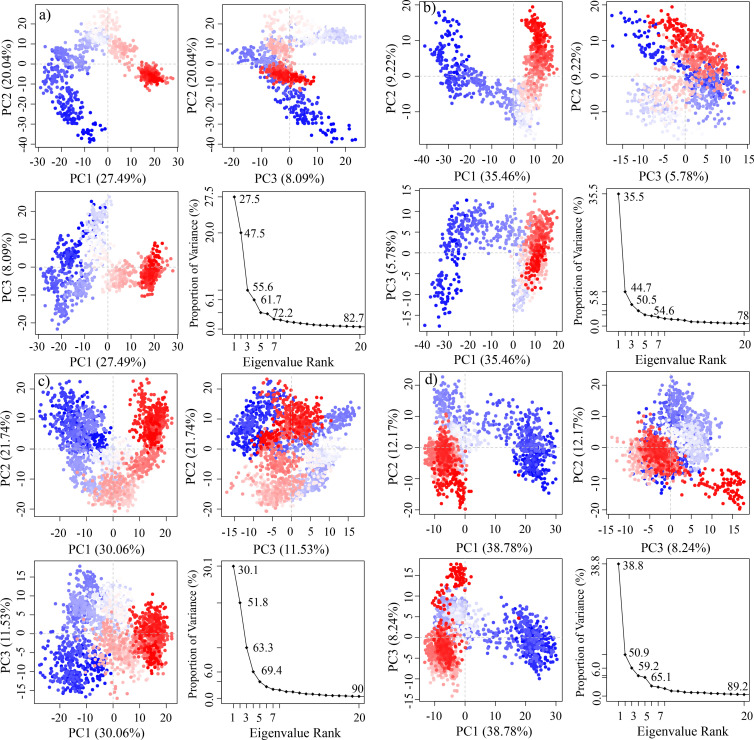
Principal component analysis of the selected complexes. a = J032-0080, b = SC13-0774, c = V030-0915, d = V006-5608.

The principal component analysis (PCA) was performed to calculate the variance percentage in protein clusters. The dominant movements were observed in the first five eigenvectors in all complexes. The eigenvalues in **J032-0080** complex were 27.5, 47.5, 55.6, 61.7, and 72.2% in the first five eigenvectors, respectively. The total variation was 82.7% while the highest fluctuations were observed in the PC1 (27.49%) (Fig 10a). Similarly, the eigen values in the first five eigenvectors of **SC13-0774** were 35.5, 44.7, 50.5, 54.6, and 64.6%, respectively. The total variation was 78%. The highest variation was observed in PC1 which recorded 35.46% fluctuations during the simulation (Fig 10b). The eigen values in the first five eigenvectors of **V030-0915** were 30.1, 51.8, 63.3, 69.4, and 79.7%, respectively. The total variation was 90%. The highest variation was observed in PC1 which recorded 30.06% fluctuations during the simulation (Fig 10c). Lastly, the total variation in the **V006-5608** complex was 89.3% with the highest fluctuation in PC1 of 38.78% (Fig 10d). The most significant movements are shown with blue regions, with intermediate motions shown by white color, while red color show the minor fluctuations [44].

#### MM/GBSA.

Molecular mechanics Generalized Born surface area (MM/GBSA) method was used to calculate the total binding free energy (ΔG_total_) for all complexes. ΔG_total_ value is usually used to estimate the stability of protein-ligand complex [45]. The lower values of ΔG_total_ indicates that the complex is more stable and vice versa. It was computed as a sum of protein-ligand complex and the difference of BACE1 protein and its ligands free energies. The total binding free energy estimated using MM/GBSA model is the outcome of the contribution of various protein-ligand interactions such as van der Waals energy (**ΔE**_**vdW**_), electrostatic energy (**ΔE**_**ele**_), **ΔG**_**GB**_ (electrostatic contribution to solvation free energy by Generalized Born). The ΔE_vdW_ contribution of **co-crystal ligand** complex was more than other complexes while the electrostatic contribution of **J032-0080** complex was more. The GB contribution showed that **J032-0080** has a higher GB value than other complexes. The total binding free energy of the complexes is shown in Table 8.

**Table 8:** The comparison of binding free energy components in selected complexes.

Energy Components	Co-crystal	J032-0080	SC13-0774	V030-0915	V006-5608
**ΔE**_**vdW**_	−55.99	−28.80	−44.14	−51.78	−54.34
**ΔE**_**ele**_	−15.60	−257.93	−189.33	−200.14	−185.79
**ΔE**_**GB**_	45.74	262.44	214.89	222.82	222.17
**ΔE**_**surf**_	−7.40	−4.59	−5.37	−6.35	−7.22
**ΔG**_**gas**_	−71.60	−286.74	−233.47	−251.93	−240.13
**ΔG**_**solv**_	38.34	257.85	209.51	216.46	214.95
**ΔG**_**total**_	−33.26	−28.88	−23.95	−35.47	−25.18

## 4. Discussion

The results of this study provide a thorough examination of novel compounds found to inhibit BACE1, a crucial target in the management of Alzheimer’s disease (AD). BACE1 has long been known to have a significant role in the pathophysiology of the disease as the enzyme that cleaves amyloid precursor protein (APP) and produces amyloid-beta (Aβ) peptides, which assemble form plaques that are characteristic of AD. The finding of BACE1 inhibitors has been the subject of numerous recent investigations; although a number of compounds have been identified, many of them have significant challenges with efficacy, selectivity, and safety [46–49]. By combining structural refinement, pharmacophore-based screening, molecular docking, and *in silico* ADMET (absorption, distribution, metabolism, excretion, and toxicity) predictions, the current study offers a novel approach that offers a more focused and refined way to find promising candidates

The study started by addressing the shortcomings of earlier research, which frequently depended on inadequate or insufficient structural representations of BACE1. The researchers refined the structure of the enzyme to produce a more realistic representation for upcoming computational investigations by identifying and modeling missing loop sections using the Protein Data Bank (PDB) structure of BACE1. This degree of structural refinement is essential because minor errors in protein structure can lead to incorrect predictions of binding sites and interactions, which may eventually cause compounds to fail in subsequent experimental phases. Further ensuring the accuracy and integrity of the BACE1 model is the use of quality assessment tools like ERRAT, Verify 3D, MolProbity, and PROCHECK in conjunction with loop modeling tools like Modeller and structure refinement tools like GalaxyRefine. An essential first step in enhancing the dependability of upcoming virtual screening initiatives is the selection of the second model on the basis of improved quality criteria.

When compared to earlier studies, this study’s strategy to create a pharmacophore hypothesis stands out. Earlier studies frequently relied on broad screening of compound libraries, whereas the current work used a more specific pharmacophore-based approach. By focusing on seven distinct properties within the BACE1 binding region, the authors were able to identify compounds that closely matched these traits, providing a more selective approach to inhibitor design. This methodology improves on previous studies, which usually used broader molecular docking approaches without such a concentrated approach to pharmacophore modeling, resulting in the inclusion of less specific or useless drugs. By establishing a clear pharmacophore hypothesis and screening compounds from the ChemDiv database’s CNS library, the study successfully identified a set of hits that were ranked based on their phase screen score, taking into account critical factors such as volume score, RMSD site matching, and vector alignment.

The findings of the molecular docking experiments emphasize the current approach’s uniqueness and strength. A variety of binding interactions were discovered, with numerous drugs forming multiple hydrogen bonds with BACE1’s critical active site residues. These interactions are critical to the stability and specificity of the protein-ligand complex, as evidenced by binding affinities greater than -5 kcal/mol for the selected hits. The docking data show that the drugs occupy the same space within the predicted binding pocket, indicating similar binding mechanisms and potential as competitive inhibitors of Aβ production. This is in contrast to other prior research, which did not capture such particular binding information, occasionally neglecting the importance of single residue interactions in favor of larger binding profiles.

The thorough bioactivity study of the present work, which forecasted the possible activity of the discovered compounds against a variety of human receptors, is one of its main advantages. Given that many lead compounds in drug discovery begin with moderate activity and then undergo additional optimization, the compounds’ moderate bioactivity ratings, which ranged from -5 to 0, suggest that they have promising drug-like qualities. The ADMET analysis further supported the compounds’ drug-likeness by indicating that all of the chosen compounds had favorable features, including appropriate molecular weights, low cLogP values, and TPSA values that indicate good oral bioavailability. The compounds also demonstrated permeability across the blood-brain barrier (BBB), which is important for the development of drugs that target the central nervous system (CNS), particularly Alzheimer’s disease.

The use of molecular dynamics (MD) simulations in this work to evaluate the stability of protein-ligand complexes is a noteworthy development in terms of novelty. The incorporation of MD simulations enables the assessment of the dynamic behavior of the protein-ligand interactions over time, whereas prior research on BACE1 inhibitors has generally concentrated on molecular docking and binding affinities. The complexes stayed stable during the simulation period, according on the RMSD and RMSF analyses from the MD simulations. The RMSD values showed that the protein and ligand had only minor conformational changes. This shows that the selected chemicals bind to BACE1 without causing major structural changes, which is a positive indicator of the interactions’ stability in a physiological setting. Furthermore, the radius of gyration (Rg) measurement revealed that ligand binding had no substantial effect on the compactness of the protein structure, validating the complexes’ durability.

The use of principle component analysis (PCA) to study variations in protein dynamics revealed additional insights into the nature of the binding interactions, indicating that the chosen ligands cause large fluctuations in protein structure. This shows that these chemicals may impact BACE1’s flexibility in a way that inhibits its enzymatic activity while maintaining the protein’s overall structural stability. The MM/GBSA calculations, which estimate binding free energy, confirmed the assumption that these drugs have robust and stable interactions with the BACE1 enzyme. Lower ΔGtotal values indicated more favorable binding.

Overall, the current study stands out for taking a comprehensive and multifaceted approach to Alzheimer’s disease drugs discovery. While many recent studies have focused on identifying BACE1 inhibitors using traditional high-throughput screening or less refined computational methods, this work combines advanced structural modeling, pharmacophore-based screening, molecular docking, and MD simulations to provide a more comprehensive assessment of potential inhibitors. This combination of approaches provides a strong strategy for discovering lead compounds with excellent specificity, stability, and drug-likeness. Furthermore, the study emphasizes the potential of these compounds to not only inhibit BACE1, but also to lay the groundwork for future Alzheimer’s therapy optimization and development. The discovery of molecules that can pass the blood-brain barrier, as well as their favorable pharmacokinetic profiles, increases their therapeutic potential, making them intriguing candidates for future Alzheimer’s disease treatments.

## 5. Conclusion

This work investigated small compounds as inhibitors of BACE1, a crucial enzyme involved in the development of amyloid cerebrovascular disease, using molecular modeling and simulation techniques. Four small compounds J032-0080, SC13-0774, V030-0915, and V006-5608 were found by virtual screening, docking analysis, and molecular dynamics simulations, demonstrating excellent binding affinity and stability inside the active region of BACE1. These inhibitors had favorable pharmacokinetic and drug-like qualities, making them promising candidates for further study. Our findings emphasize the therapeutic potential of these small compounds, pointing to an approach for treating amyloid-related cerebrovascular disorders, specifically Alzheimer’s disease. Further experimental validation is needed to confirm their efficacy in biological systems.
